# Scientific Items

**Published:** 1881-04-20

**Authors:** 


### SCIENTIFIC ITEMS.

**Recent Progress in Photography.**—Some two years ago the whole photographic world was startled by the announcement that Mr. C. Bennet could obtain pictures in the camera in but a fraction of the time necessary with any process extant up to that time, and he demonstrated the fact to the satisfaction of every one. The process he used was founded on that of Dr. Maddox, and consisted of bromide of silver in a state of minute subdivision held in suspension in a viscous solution of gelatine. The rapidity was gained by keeping the gelatine containing the suspended matter in a liquid condition for several days.

In the recent photographic exhibition in Pall Mall we saw examples of the rapidity of which the gelatine process is capable. A train going 60 miles an hour was fairly expressed on the photographic plate, there being a sharpness of image which was truly marvelous. The 1-150th part of a second is a short interval of time, yet in such a time the picture was taken. Again, one of the pictorial series of interest of the year was a series of views on the river Thames by Mr. Mayland. The silent highway is sparkling with motion, shipping and steamers at full speed being rendered sharply and clearly—in fact, the idea of motion is conveyed by the foaming tracks and curling waves which they make in their passage.

Drawing-room photography by amateurs now becomes a possibility and a practicability which before it was not, and in one remarkable picture of the year we have a dark interior of a room, and a portrait of a charming model secured on one plate in 25 seconds of time. In the old days such a combination would have been impossible, except by printing from two or more negatives. Mr. H. P. Robinson, whose artistic work is so well known and so greatly admired, has thus scored a success in adapting the process to his peculiar style of pictures. In science, however, the rapid plates have proved of more than yeoman service.

One of the most marvelous feats, however, of photography is the portrayal of the motion of trotting, cantering, and galloping horses by Mr. Muybridge, in America, in which he shows changes in the position of every limb during minute fractions of a second, and this settles certain points which have exercised the minds of certain eminent physiologists.

If we quit the more rapid process and come once more back to the old collodion process, we find that it has also done good service in scientific research. In the Bakerian Lecture of the Royal Society for the past year, Captain Abney has shown how the sensitive compound bromide of silver can be so modified as to cause radiations of very low wave-length to impress themselves on the photographic plate, and in a map he gives the Fraunhofer lines which exist in the solar spectrum far down below the visible red rays of the spectrum. In the prints as usually supplied, the images, which are built up of minute particles of silver, gradually fade and become worthless.—*London Times.*

**Man in America.**—Prof. Flower, in a recent lecture on the "Anatomy of Man," before the Royal College of Surgeons, London, discussed at some length the question of his origin on the American continent. Till recently, opinions on the early peopling of America had been divided between the views that the inhabitants of this continent were a distinct indigenous people, and therefore not related to those of any other land; and that they were descended from an Asiatic people who, in comparatively recent times, passed into America by the way of Behring Strait, and thence spread gradually over the whole continent.

These theories have had to undergo considerable modifications, in consequence of the discovery of the great antiquity of the human race in America, as well as in the Old World. The proof of this antiquity rests upon the high and independent state of civilization which had been attained by the Mexicans and Peruvians at the time of the Spanish conquest, and the evidence that that civilization had been preceded by several other stages of culture, following in succession through a great stretch of time.

The antiquity of this quasi-historical period is, however, entirely thrown into the shade by the evidence now accumulating from various parts of North and South America, that man existed on the Western Continent, and under much the same conditions of life, using precisely similar weapons and tools, as in Europe during the Pleistocene or Quarternary period, and even perhaps farther back in time. Recent paleontological investigations show that an immense number of forms of terrestrial animals, that were formerly supposed to be peculiar to the Old World, are abundant in the New.

Taking all circumstances into consideration, it is quite as likely that Asiatic man may have been derived from America as the reverse, or both may have had their source in a common center, in some region of the earth now covered with sea.—*Pop. Science Monthly.*

**Germs of Disease in Water.**—Prof. Huxley, in a recent discussion of a paper by Dr. Tidy on water for dietetic purposes, said that diseases caused by what people not wisely call germs are produced invariably by bodies of the nature of bacteria. These bodies could be cultivated through twenty or thirty generations, and then, when given under the requisite conditions, would invariably cause their characteristic disease.

They can be sown and will thrive in Pasteur's solution, just as cress or mustard in the soil; and, if a drop of this solution were placed in a gallon of water, Prof. Roscoe thinks it doubtful if there is any known method by which its constituents could be estimated. One drop of it would be capable of exciting a putrefactive fermentation in any substance capable of undergoing that fermentation. The human body may be considered as such a substance, and we may conceive of a water containing such organisms which may be as pure as can be as regards chemical analysis, and yet be, as regards the human body, as deadly as prussic acid.

This is a terrible conclusion, but it is true.—*Pop. Science Monthly.*

